# Targeting metabolic rewiring might decrease spread of tumor cells: Mitochondrial tRNA modifications promote cancer metastasis

**DOI:** 10.1038/s41392-022-01205-6

**Published:** 2022-10-08

**Authors:** Maik Luu, Alexander Visekruna

**Affiliations:** 1 Lehrstuhl für Zelluläre Immuntherapie, Medizinische Klinik und Poliklinik II, Universitätsklinikum Würzburg, Würzburg, Germany; 2 Institute for Medical Microbiology and Hygiene, Philipps-University, Marburg, Germany

**Keywords:** Metastasis, Metastasis

In a recent study published in *Nature*, Delaunay and colleagues demonstrate that tRNA modifications in mitochondria have the potential to affect the synthesis of mitochondrial proteins involved in oxidative phosphorylation (OXPHOS), which drives the invasive spread of cancer cells. The inhibition of one particular RNA-modifying enzyme was sufficient to prevent the cancer cell invasion and dissemination.^1^

**Fig. 1:**
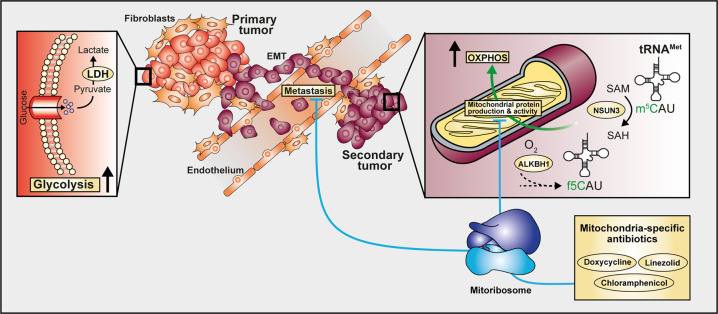
Mitochondrial tRNA modifications are involved in development of metastases by regulating metabolic plasticity of invasive tumor cells. Due to shortage of oxygen and nutrients in the tumor microenvironment of primary tumors, the tumor cells utilize glycolysis instead of OXPHOS for their energy supply. The metabolic switch from glycolysis to OXPHOS occurs when invasive tumor cells leave the primary tumors and settle in a new area (secondary tumors). Specific mitochondrial tRNA modifications such as m^5^C and f^5^C are introduced by the tRNA-modifying enzymes NSUN3 and ALKBH1, respectively. These modifications are essential for optimal protein synthesis of enzymes involved in mitochondrial respiration. In mice bearing neck or head tumors, depletion or pharmacological inhibition of NSUN3 leads to reduction of m^5^C in mitochondrial tRNA, resulting in decreased metastases in both lymph nodes and lungs. Similar effects on metastases can be achieved by using mitochondria-specific antibiotics

Glycolysis and OXPHOS are two main metabolic pathways that support cancer cell development and progression. While primary tumors preferentially utilize glucose as a source for their energetic requirement (aerobic glycolysis, also known as the Warburg effect that is in general a hallmark of tumor metabolism), less is known about metabolic plasticity required for dissemination of cancer cells from primary tumor sites. Metastasis, which is a multistep process enabling the tumor cells to spread from primary tumors to adjacent and distinct tissues, is responsible for approximately 90% of cancer-related deaths. The metastatic development requires cancer cells to leave the primary tumors and to metabolically adopt to changing microenvironment. Figure 1.

Mitochondria, which continually undergo the highly dynamic processes of mitogenesis and mitophagy, are crucial for OXPHOS, and cancer cells are characterized by mitochondrial metabolic plasticity and heterogeneity. It is plausible to speculate that some mitochondrial metabolites might be implicated in driving oncogenesis. Mitochondria were previously proposed to be involved in both aspects of metastasis, migration, and invasion of cancer cells,^2^ however the exact underlying mechanisms and the respective roles of OXPHOS and glycolysis during metastatic transition are still unknown. Some previous studies reported that metformin, a widely used anti-diabetic drug approved for the treatment of patients with type 2 diabetes, promotes anticancer effects.^3^ Although several mechanism have been proposed for its anti-tumoral activity, the dominant effects of metformin are generally mediated by inhibiting mitochondrial electron transport chain (ETC) complex I.

The capacity of mitochondrial tRNAs to modify tumor development and metastasis has not been elucidated yet. In a recent publication in *Nature*, Delaunay and colleagues show that the specific modifications 5-methylcytosine (m^5^C) and its derivative 5-formylcytosine (f^5^C) at position 34 in mitochondrial tRNA^Met^ are required for the development of CD36-driven metastasis. The functional aspects of modifications in mitochondrial tRNAs are poorly understood. So far, 18 different types of post-transcriptional modifications introduced by tRNA-modifying enzymes have been found in 22 human mitochondrial tRNAs.^4^ It is known that all 22 tRNA species translate essential proteins belonging to respiratory chain complexes. The data from Delaunay et al. suggest that the m^5^C modification of mitochondrial tRNAs is an important prerequisite for metabolic remodeling of metastasis-initiating cells that are dependent on mitochondrial protein synthetic machinery and mitochondrial respiration. The loss of a specific mitochondrial enzyme, NOP2/Sun RNA methyltransferase (NSUN3) led to metabolic switch associated with an elevated glycolysis, an upregulation of glucose transporter 1 (GLUT1), and a limited OXPHOS, as well as a slight reduction in most tricarboxylic acid (TCA) metabolites. As a result, the invasive spread of tumor cells was strongly decreased. Interestingly, the growth of primary tumors that primarily utilize glycolysis was not affected by the loss of m^5^C modification. The authors conclude that site-specific mitochondrial tRNA modifications likely serve as a “sensor of cellular energy requirements”, which might be a novel therapeutic target for modifying metabolic plasticity needed for metastatic tumors. Moreover, the NSUN3 function as potential clinical biomarker for metastases was examined in patients with head and neck cancer with high expression of CD36. Indeed, a high cellular expression of the enzyme NSUN3 and increased m^5^C levels were highly predictive for lymph node metastases.

To gain insights into potential therapeutic aspects, the authors treated both in vitro-derived tumoroids and tumor-bearing mice with several classes of antibiotics. Of note, only antibiotics such as doxycycline, chloramphenicol, and linezolid, which are able to inhibit mitochondrial protein synthesis without affecting protein translation in the cytoplasm, reduced the number of invading leading cells in tumoroids and metastases in vivo. Thus, the pharmacological inhibition of mitochondrial translation fully recapitulated the functional loss of m^5^C formation in mitochondrial tRNAs by being able to suppress cancer cell invasion and metastatic spread. Although this novel concept suggests that specific antibiotics may be therapeutically exploited to inhibit progress of metastatic cancer, we might need a deeper understanding of such findings, as a global change of patients‘ microbiome is expected following the treatment with antibiotics. Several studies have demonstrated that antibiotic-induced disturbances in the composition of intestinal microbiota modulate T cell-mediated anti-cancer immunity.^5^ It is possible that alterations in microbiome in the gut (or even within the microbiome-harboring tumors) caused by antibiotic treatment may also impact on metastatic behavior of tumors.

In summary, Delaunay and colleagues identified the specific mitochondrial RNA modifications that are associated with invasive spread of cancer cells and poor prognosis of neck and head cancer. The specific modification of mitochondrial tRNA^Met^, known as m^5^C was crucial for the development of metastasis. Both, the blockade of the specific enzyme, NSUN3, responsible for m^5^C modification and treatment of mice with antibiotics targeting mitochondrial protein synthesis strongly decreased the invasive spread of cancer cells from the primary tumors. Thus, modifications of mitochondrial tRNAs regulate the metabolic plasticity in metastasis by supporting mitochondrial respiration of invasive cancer cells located at the periphery of primary tumors. The metabolic feature of such invasive cancer cells performing high degree of OXPHOS may be used in the future for the prevention of metastases. Although this finding represents an innovative work contributing to the therapeutic exploration of how mitochondrial RNA modifications could be targeted for cancer treatment, various aspects have remained unclarified. Since the majority of normal tissue cells rely on OXPHOS, the OXPHOS-dependent metabolic reprogramming might have various adverse effects. The function of some immune cells such NK cells and CTLs that recognize tumor cells can be impaired by inhibiting protein synthesis in mitochondria. Moreover, for some cancer patients, the high OXPHOS is associated with higher sensitivity to chemotherapy and better prognosis (the OXPHOS heterogeneity of various cancer types should also be taken into account). As high-level of f^5^C modification in mitochondrial tRNAs is present in various tissues, future work should shed light on the role of this modification and the corresponding tRNA-modifying enzyme ALKBH1 in cancer progression. Finally, the treatment with mitochondria-specific antibiotics would introduce global perturbations in gut microbiota composition, which might lead to suppression of anti-tumor immunity. Although a better understanding of mitochondrial RNA modifications may open new therapeutic opportunities, additional studies will be required to define their therapeutic potential for cancer treatment.
